# Towards Continuous and Ambulatory Blood Pressure Monitoring: Methods for Efficient Data Acquisition for Pulse Transit Time Estimation

**DOI:** 10.3390/s20247106

**Published:** 2020-12-11

**Authors:** Oludotun Ode, Lara Orlandic, Omer T. Inan

**Affiliations:** 1 Georgia Institute of Technology, School of Electrical and Computer Engineering, Atlanta, GA 30332, USA; inan@gatech.edu; 2 Embedded Systems Laboratory (ESL), EPFL, 1015 Lausanne, Switzerland; lara.orlandic@epfl.ch

**Keywords:** pulse transit time, blood pressure, electrocardiogram, photoplethysmogram, seismocardiogram, gyrocardiogram, compression, Huffman coding, UART flow control

## Abstract

We developed a prototype for measuring physiological data for pulse transit time (PTT) estimation that will be used for ambulatory blood pressure (BP) monitoring. The device is comprised of an embedded system with multimodal sensors that streams high-throughput data to a custom Android application. The primary focus of this paper is on the hardware–software codesign that we developed to address the challenges associated with reliably recording data over Bluetooth on a resource-constrained platform. In particular, we developed a lossless compression algorithm that is based on optimally selective Huffman coding and Huffman prefixed coding, which yields virtually identical compression ratios to the standard algorithm, but with a 67–99% reduction in the size of the compression tables. In addition, we developed a hybrid software–hardware flow control method to eliminate microcontroller (MCU) interrupt-latency related data loss when multi-byte packets are sent from the phone to the embedded system via a Bluetooth module at baud rates exceeding 115,200 bit/s. The empirical error rate obtained with the proposed method with the baud rate set to 460,800 bit/s was identically equal to 0%. Our robust and computationally efficient physiological data acquisition system will enable field experiments that will drive the development of novel algorithms for PTT-based continuous BP monitoring.

## 1. Introduction

### 1.1. Rationale

High blood pressure is a high risk factor for cardiovascular diseases (that cause 7.1 million deaths annually) [1] and effective BP monitoring may potentially decrease mortality and improve a patient’s quality of life by reducing hospitalization [2]. We are currently developing a wearable system that would, in contrast to traditional cuff-based methods, enable 24-h continuous ambulatory BP monitoring. We envision an unobtrusive device based on pulse transit time (PTT) methods that is comprised of wearable seismocardiogram (SCG) and gyrocardiogram (GCG) for proximal timing, reflectance-mode photoplethysmogram (rPPG) for distal timing, and an electrocardiogram (ECG) that is used as a timing reference. Studies suggest that PTT—the time that it takes for an arterial pressure wave to travel between two locations—is correlated with BP and can therefore be used to obtain BP measurements [3,4].

Data measured with these sensing modalities would ideally be preserved for further analysis by a clinician, which can be achieved either by storing the data locally on a Secure Digital (SD) card or by transmitting them over a wireless network to, for instance, a Bluetooth-enabled smartphone. The latter is preferable because it minimizes the burden placed on the user for reliably transferring data from the wearable device. In addition, computationally demanding tasks such as PTT estimation may be delegated to the smartphone (or to the cloud with the smartphone acting as a conduit), which means that the wearable may be designed to use low-cost components with very modest computational resources. A smartphone acting in an Internet gateway role may also be used to deliver over-the-air firmware updates and enable interesting applications such as customized sensing. The authors of [5] developed an Android application with real-time data streaming capabilities for PTT estimation; however, their system samples 8-bit ECG at 300 Hz and a single channel finger PPG at 75 Hz. The reported sampling rates and resolution are likely insufficient for accurate PTT detection (typical sampling rates reported in existing literature are in the 1–2 kHz range [6,7]). Moreover, an ECG sensor and a PPG sensor alone can only be used to measure pulse arrival time, which is not a reliable predictor of BP [8].

There are a few limitations associated with Bluetooth communications that need to be addressed before it can be reliably used for PTT sensing. First, the severe bandwidth constraints of Bluetooth communication over a universal asynchronous receiver–transmitter (UART) interface restricts the maximum sustained data rate to 45 kB/s (a two-bit overhead (for the start and stop bits) is incurred for every byte transmitted over UART, so we adopt bit/s as the unit for the raw data rate and B/s for the usable data rate—to distinguish between both quantities) for the low-power MCU and Bluetooth module pair used in this work, which is significantly lower than the achievable 2 mB/s (or higher) SD card speeds over a serial peripheral interface (SPI). Second, the inherently lossy nature of wireless communications, which results in dropped packets and missing data, poses a significant challenge for PTT applications that require accurate detection of fiducial points on the acquired waveforms. Finally, the active mode current consumption of the Bluetooth device used in this work can be as high as 26 mA—a relatively large value for wearables where battery power is at a premium. All of the aforementioned drawbacks can be mitigated by data compression via source coding. Data compression simultaneously minimizes bandwidth usage and current consumption because the Bluetooth radios spend less time in active mode. Furthermore, the bandwidth savings can be used to implement an application layer that enables lossless communications over the lossy interface.

### 1.2. Data Compression Background

Data compression methods can be broadly categorized, based on their ability to perfectly recover the original data from its compressed form, into lossy and lossless compression. Several authors have developed lossy compression algorithms for physiological data such as ECG [9,10] and electroencephalograms [11], and most have reported very high compression ratios but with varying degrees of compromises made with regards to the reconstructed signal quality. The percent root-mean square difference (PRD) is often used a metric for quantifying the signal reconstruction fidelity [12,13]. Lossy compression is certainly a sensible approach for cardiac analysis methods, such as heart rate monitoring, that depend on aggregate morphological features of the measured waveforms. In contrast, the singular focus in PTT sensing is on accurately identifying the fiducial locations on the cardiac signals of interest. In particular, a reconstructed PPG waveform with a smeared pulse foot, but with the rest of the waveform perfectly reconstructed, would likely have a low PRD although the smearing of the pulse foot may render the waveform virtually useless for PTT estimation. Moreover, the type of distortion is not known a priori, so lossy compression is not ideal for PTT sensing applications.

Lossless compression algorithms typically implement an estimator for the underlying signal and encode the residual between the true signal and the estimate with an entropy reducing algorithm [14]. The residual should, in theory, have a much smaller dynamic range in comparison to the true signal. Estimators that are commonly used for physiological data compression include transform-based methods such as wavelets [15,16] and linear predictors used in linear predictive coding [17]. These methods are inappropriate for low-power computing platforms because of their relatively high computational cost. A more promising method is differential pulse code modulation (DPCM) [18,19], where the estimate of the current sample is simply the previous sample, therefore the residual corresponds to the first backward difference. The DPCM method relies on the observation that adjacent samples in naturally occurring signals are highly correlated [19].

Popular entropy reducing algorithms include Huffman coding [20] and arithmetic coding. Arithmetic coding provides higher compression ratios [21], but Huffman coding is a more attractive choice for this application because it only requires a table lookup, which can be implemented with hash tables that support very fast and efficient queries. Huffman codes are variable-length codes that assign fewer bits to frequently occurring symbols and longer bit sequences to less frequent ones. The main challenge associated with implementing the Huffman coding algorithm on a low-power MCU is its prohibitive storage requirement. The reason is that, for lossless compression, every possible symbol in the dictionary must be explicitly stored in the compression table, which implies that *M*-bit data would require a table with $2^{M}$ entries. Clearly, $O(2^{M})$ space complexity does not scale well to low-power platforms with memory capacities typically in the kB range. Furthermore, the size of the underlying data types required to store the codes may increase because longer bit sequences are used to encode low probability symbols.

**Figure 1:**
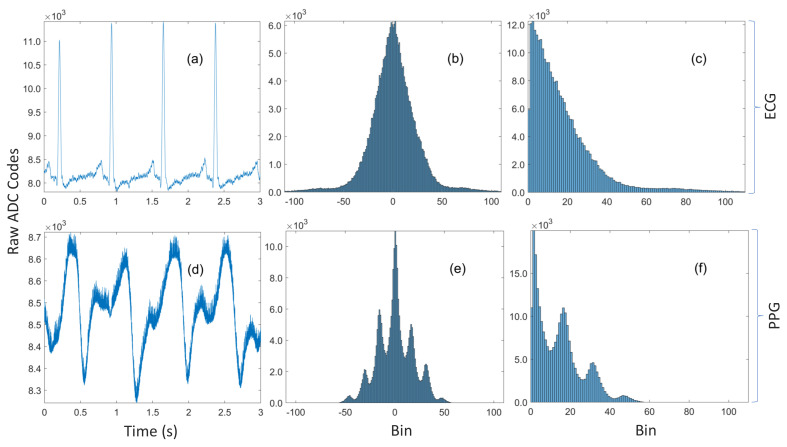
(**a**) A three-second trace of ECG data recorded during device prototyping. (**b**) The unimodal histogram of residuals for the entire ECG dataset with 260k samples. (**c**) The residuals are approximately symmetric, so a sign bit can be transmitted along with the encoded magnitude. (**d**) A three-second trace of noisy rPPG data recorded simultaneously with the ECG. (**e**) The multimodal histogram of residuals for the rPPG. (**f**) The histogram of magnitudes of the residuals for the rPPG.

One method that has been proposed for minimizing the Huffman table size is the so-called optimally selective Huffman coding scheme [22], which explicitly encodes the *k* most frequent symbols (where *k* is a user-defined parameter), while other symbols are transmitted in their raw form. A preceding bit is used to indicate whether the succeeding bits are encoded or raw data. Without loss of generality, let $k=2^{m}$, where $m\in Z_{\geq 0}$; if the *k* symbols are uniformly distributed, then the average code length for the *k* symbols is given by the maximal entropy, which is $\log_{2}\left(k\right)=m$ [23]. However, if the $2^{m}$ symbols can be grouped together and there exists a natural ordering among them, then there is the trivial bijection between the set of *k* symbols and the index set $\left\{0,1,\ldots ,2^{m}-1\right\}$ mapping the symbols to their respective positions, where each index has an *m*-bit binary representation. Therefore, an identical compression ratio can be achieved—without the use of a compression table—by transmitting the symbol’s index within the group instead of its Huffman code. This example, which highlights the main drawback of the algorithm, may seem to be somewhat contrived, however analysis of the data in Figure 1 reveals that this is not the case. Figure 1c is the histogram of magnitudes of residuals for 14-bit ECG data recorded during device development. Although the first 16 $\left(2^{4}\right)$ bins are not exactly uniformly distributed, their Huffman codes (computed with MATLAB and provided in Appendix A.3) are all 4-bits long. Therefore, residuals in bin 0 can be encoded as 0 $\left(0000_{b}\right)$, bin 1 as 1 $(0001_{b}),\ldots$, and bin 15 as 15 $\left(1111_{b}\right)$ without increasing the average code length of the 16 symbols. It should be noted that this behavior served as the primary rationale for the algorithm developed in this work. Another limitation is that it is highly susceptible to overfitting—a problem shared with other greedy algorithms. The distribution of the finite dataset that the codes are computed from may not be representative of the true distribution. For example, PPG amplitudes are well-known to be highly dependent on contact/hydrostatic pressure and perfusion (amongst other factors) [24,25,26]. Therefore, its empirical distribution is dependent on those parameters and the data used to compute the codes may not be consistent with the data recorded later. Nevertheless, optimally selective Huffman coding is just a special case of the algorithm proposed in this work and this special case is selected if it is indeed optimal in the context that is defined shortly.

Huffman prefixed coding (not to be confused with the prefix-free property of Huffman codes) is an alternative strategy for constructing tables from potentially large dictionaries by grouping the symbols into equiprobability classes with Huffman codes computed for those probability classes alone [27]. The code for a symbol is subsequently formed by concatenating the Huffman code for its probability class with the index of the symbol within that class. Although not explicitly acknowledged, this method was used by Pahlm [28], where the groups are of the form {0} (the special case) and the nonuniform half-open intervals [$2^{m-1},2^{m}$) for m≥1 (the general case). This grouping implicitly makes very strong but not necessarily correct assumptions about the nature of the distribution. Specifically, it assumes that the distribution decays rapidly but that it is approximately piecewise constant within each of the intervals. That assumption is definitely reasonable for the unimodal ECG distribution in Figure 1c, but not so for the noisy PPG data in Figure 1d, which has the multimodal distribution shown in Figure 1f. Moreover, the algorithm has no tuning parameters, so the one-size-fits-all approach is unlikely to be optimal for every possible waveform and sensing modality. Furthermore, it treats the symbol ‘0’ as a special case but it can be observed in Figure 1c,f that ‘0’ is not always the most probable symbol.

### 1.3. Contributions

We developed an end-to-end system that will be used for conveniently recording high-throughput physiological data in a mobile setting, which will guide algorithm development for PTT-based ambulatory BP monitoring. To that end, we implemented a breadboard prototype that incorporates off-the-shelf and custom analog front-ends for sensing rPPG, SCG, GCG, and ECG. We also developed a software suite comprised of firmware running on a low-power MCU and an Android application for acquiring data in real-time, a desktop application for recovering data offline, and a back-end web application for storing de-identified subject metadata.

In addition, we propose a compression algorithm that is based on optimally selective Huffman coding and Huffman prefixed coding, where compact Huffman tables are computed from a subset of data grouped into probability classes. Furthermore, we developed a fast algorithm for learning the tables in a computationally efficient manner, and we demonstrate how to leverage the computational capabilities of modern smartphones such that the compression tables can be computed in real-time from streaming data. We compare compression ratios obtained with the proposed method to those obtained with Huffman tables computed from complete data. We also demonstrate how this method may be used to acquire and transmit physiological data at a rate higher than the theoretical channel capacity of the Bluetooth module.

Finally, we addressed the need for lossless data communications by implementing: (1) a hybrid hardware–software UART flow control method to eliminate MCU interrupt-latency related errors; and (2) an acknowledgment-based (ACK-based) lossless communications protocol for reliably transmitting sensor data from the wearable to a smartphone, and compression tables and messages in the reverse direction.

## 2. Materials and Methods

### 2.1. Algorithm

In this subsection, we first present the proposed algorithm and its associated grouping strategy. Next, we derive a computationally efficient algorithm for learning the hyperparameters of the proposed algorithm and the corresponding compression table.

#### 2.1.1. Encoder

**Figure 2:**
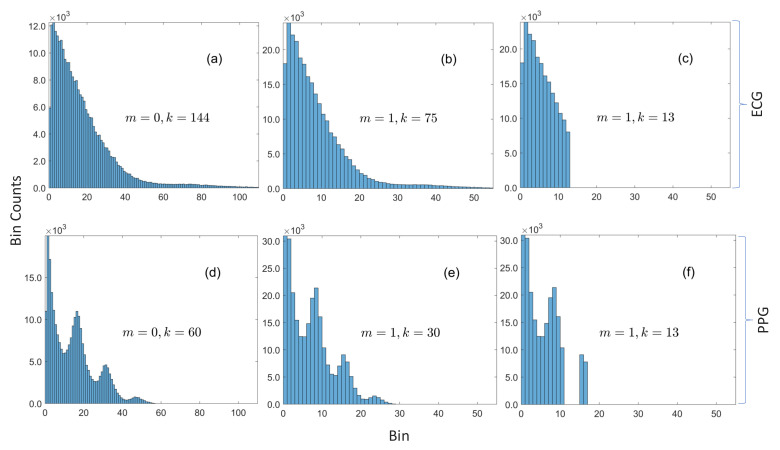
Histograms of magnitudes of residuals for ECG and PPG. (**a**) ECG with bin width = 1 is sparse (with respect to the underlying dictionary of $2^{14}$ possible values) with only 144 bins with nonzero counts. (**b**) The number of bins with nonzero counts is approximately halved when the bin width is increased to 2. (**c**) The 13 most frequent bins are in contiguous slots. (**d**) PPG with bin width = 1 is also sparse with only 60 bins with nonzero counts. (**e**) The number of bins with nonzero counts is halved when the bin width is increased to 2, and the distribution still retains the same multimodal form. (**f**) The 13 most frequent bins are not all in contiguous slots, which demonstrates how the proposed method can sift out modes in multimodal distributions.

The histograms in Figure 2a,d provide the rationale for the proposed method. It can be observed that the bin counts are approximately constant over a *small* neighborhood (how small the neighborhood is will be discovered from data with an approach that is subsequently described). If the neighborhoods are restricted to be bins of the zero-aligned histogram with bin widths of the form $2^{m}$, where $m\in Z_{\geq 0}$, then the probability class of a residual r[n] is $\left\lfloor \frac{\left|r[n]\right|}{2^{m}}\right\rfloor$ and its index within the class is $|r\left[n\right]|\;\mathrm{mod}\;2^{m}$. This grouping strategy is convenient because, for *M*-bit data, the (M−m) most significant bits of the magnitude of the residual correspond to the sample’s probability class while the *m* least significant bits is the residual’s index within the probability class, which greatly simplifies the encoder and the decoder. It is worth noting that this method is, to some extent, similar to the content-adaptive Golomb–Rice coding algorithm that was developed for compressing ECG by Tsai [29] because both techniques encode the lower *m* bits in binary. The key differences are: (1) the Golomb–Rice algorithm unary encodes the upper (M−m) bits; and (2) they learn *m* with a windowing method that is determined by the QRS complex of the ECG. Our approach is more general and can be used for different physiological sensing modalities without any modification, because it does not rely on any morphological features of the waveform in order to perform compression. Moreover, their overall system is more complex than the one proposed in this paper, which may significantly reduce sampling rates (with similar hardware, they recorded twelve channels of 11-bit ECG at 600 Hz, compared to the 3200 Hz sampling rate for ten 14- and 16-bit channels in this work).

**Figure 3:**
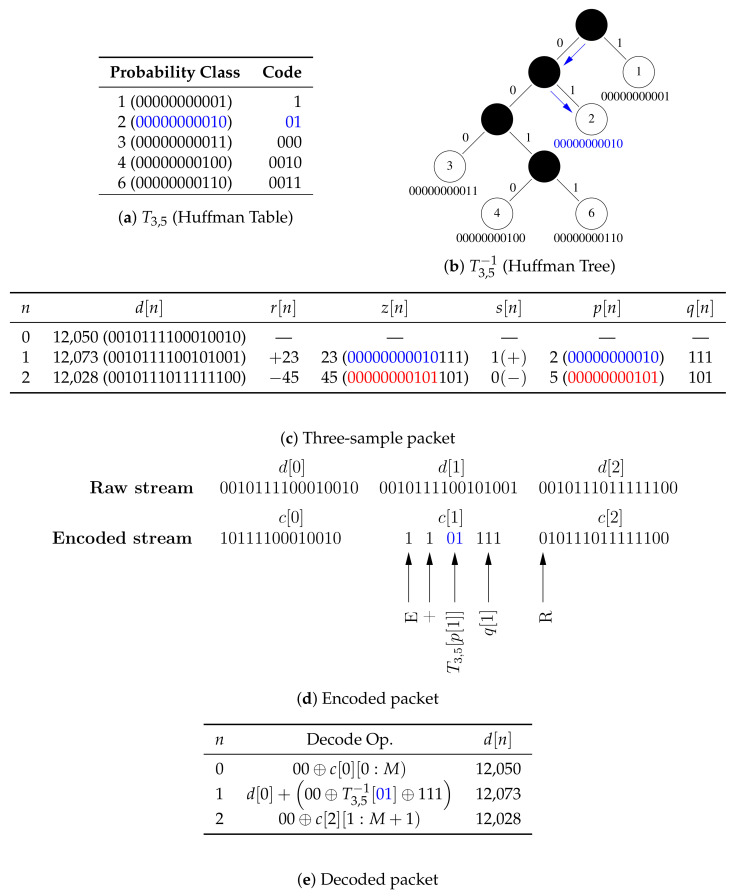
A simulated example of encoding and decoding a three-sample packet with a 14-bit single data channel. (**a**) The compression table stored on the MCU (M=14,m=3,k=5), which maps the 11 upper bits of the magnitude of residuals to Huffman codes. (**b**) The tree stored on the smartphone for decoding Huffman codes. (**c**) d[0] is sent in its raw form so that packets are self-contained and can be decoded out of order, d[1] is encoded because 2 is in the compression table, and d[2] is transmitted in its raw form because 5 is not in the compression table. (**d**) An illustration of the steps for encoding packets. (**e**) An illustration of the steps for decoding packets. It should be noted that: (1) the two uppermost unused bits are removed by the encoder and replaced by the decoder; and (2) $\beta [n_{1}:n_{2})$ denotes values of bits $n_{1},n_{1}+1,\ldots ,n_{2}-1$ in the bit sequence β.

As with the optimally selective Huffman coding algorithm, we compute Huffman codes for only a subset of the data, but, instead of making the table size a fixed parameter, we specify a search range $\left\{k_{1},k_{1}+1,\ldots ,k_{2}\right\}$ of acceptable table sizes and we compute tables for probability classes as opposed to residuals. Figure 2c,f shows the selected subsets when m=1 and k=13, and it can be observed that this grouping method works well with unimodal and multimodal distributions because it is able to sift out three out of four modes in Figure 2f. The encoder is explicitly given in Algorithm 1, but the decoder is omitted for brevity (because it can be inferred from the encoder). Nevertheless, both are illustrated by the simulated example given in Figure 3. The encoder for an *M*-bit sample d[n] checks if the Huffman code for the sample’s probability class is in the compression table $T_{m,k}$, where *m* is the bin width parameter and *k* is the size of table. If so, the sample’s code is constructed by concatenating the encoded data indicator, the sign indicator for the residual, the Huffman code for the residual’s probability class, and its index within the class. If the code for the probability class is not in the table, the code is formed by concatenating the raw data indicator with the raw sample—with any unused leading bits removed.
**Algorithm 1.** encode.**Input:**$d[n],d[n-1],m,M,T_{m,k}$**Output:**c[n]1:E←12:R←03:r[n]←d[n]−d[n−1]4:z[n]←|r[n]|5:$p_{m}^{D}\left[n\right]\leftarrow \left\lfloor \frac{z[n]}{2^{m}}\right\rfloor$6:**if**$p_{m}^{D}\left[n\right]\in \mathrm{dom}\left(T_{m,k}\right)$**then**7:$\;q\left[n\right]\leftarrow z\left[n\right]\;\mathrm{mod}\;2^{m}$8:   **if** r[n]≥0 **then**
9:    s[n]←110:  **else**11:    s[n]←012:  **end** **if**13:$\;c\left[n\right]\leftarrow^{\left(1\right)}E{⊕}^{\left(1\right)}s\left[n\right]⊕T_{m,k}\left[p_{m}^{D}\left[n\right]\right]{⊕}^{\left(m\right)}q\left[n\right]$14:**else**15:$\;c\left[n\right]\leftarrow^{\left(1\right)}R{⊕}^{\left(M\right)}d\left[n\right]$16:**endif*****Comments:**(1) $\left|\cdot \right|$ Denotes the magnitude or cardinality (size) of its given argument when applied to a scalar or a set, respectively; (2)* ⊕ *Denotes bitwise concatenation; and (3) Leading superscript explicitly indicates the size (in bits)*


#### 2.1.2. Hyperparameter Search

The primary task of computing $T_{m,k}$ from data involves learning *m* and *k* because, given those parameters, $T_{m,k}$ can be computed in a trivial manner. To that end, we search for the hyperparameters with model validation—a standard supervised machine learning method. Specifically, we partition the time series data S≜s[0],s[1],…,s[J−1], which are the sensor data for a particular channel, into equal-length non-overlapping subsequences T and V that are labeled *training* and *validation* data, respectively (with the samples in T preceding those in V). We then proceed to learn $T_{m,k}$ from T by evaluating its performance, via a chosen objective function, on unseen data V, which is done to minimize the risk of overfitting. It is reasonable to expect a high degree of similarity between the *k* probability classes from which $T_{m,k}$ is computed and the empirical distribution of the validation data. The Kullback–Leibler divergence is often used to evaluate the similarity (or more accurately, the dissimilarity) between two distributions [30]. However, a high degree of agreement between both distributions is only a likely artifact and not the desired goal, which is to learn a table that maximizes the compression ratio. The compression ratio, which can be maximized by minimizing the total number of bits, is therefore chosen as the objective function. The hyperparameter search problem can then be expressed as the optimization:

(1) $$m^{*},k^{*}=\underset{0\leq m<M,k_{1}\leq k\leq k_{2}}{arg min}\Phi \left(V;m,M,T_{m,k}\right)$$

where

(2) $$\Phi \left(V;m,M,T_{m,k}\right)≜\sum_{n=0}^{N-1}\mathrm{len}\left(\mathrm{encode}\left(v\left[n\right];v\left[n-1\right],m,M,T_{m,k}\right)\right)$$

and len(β) denotes the length (in bits) of the bit sequence β. It should be noted that Equation (2) assumes that there is a fictitious sample v[−1]=0.

A brute-force search is used to implement the optimization program defined in Equation (1) because there is no closed-form expression for evaluating Equation (2). The computational cost of Φ for any {m,k} pair is O(N), which is far from ideal for a real-time implementation when *N* is large. However, the inherent structure in Algorithm 1 can be exploited to derive a more computationally efficient algorithm that may be used to learn $\left\{m^{*},k^{*}\right\}$ from arbitrarily large streaming datasets.

The key observation that can be derived from Algorithm 1, for the purpose of accelerating the search, is that, given m,M, and $T_{m,k}$, the code length for any sample v[n] is completely specified by the probability class of its residual

(3) $$p_{m}^{V}\left[n\right]≜\left\lfloor \frac{\left|v[n]-v[n-1]\right|}{2^{m}}\right\rfloor$$

that is,

(4) $$\Phi \left(V;m,M,T_{m,k}\right)=\sum_{n=0}^{N-1}\phi \left(p_{m}^{V}\left[n\right];m,M,T_{m,k}\right)$$

where

(5) $$\phi \left(\alpha ;m,M,T_{m,k}\right)≜\left\{\begin{array}{cc} 2+\mathrm{len}(T_{m,k}\left[\alpha \right])+m, & \mathrm{if}\;\alpha \in \mathrm{dom}(T_{m,k})\\ 1+M, & \mathrm{otherwise} \end{array}\right.$$

Moreover, $p_{m}^{V}\left[n\right]$ is not necessarily unique in V—especially when *N* is large. Therefore, the terms with common arguments in Equation (4) can be grouped together into the compact form given below

(6) $$\begin{array}{cc} \sum_{n=0}^{N-1}\phi \left(p_{m}^{V}\left[n\right];m,M,T_{m,k}\right) & =\sum_{x\in \overset{N-1}{\underset{n=0}{⋃}}\left\{p_{m}^{V}\left[n\right]\right\}}\left(\sum_{j=0}^{N-1}1_{\left\{p_{m}^{V}\left[j\right]=x\right\}}\right)\phi \left(x;m,M,T_{m,k}\right) \end{array}$$

(7) $$\begin{array}{cc} & =\sum_{x\in \mathrm{supp}(H_{m}^{V})}H_{m}^{V}\left[x\right]\phi \left(x;m,M,T_{m,k}\right) \end{array}$$

where $H_{m}^{D}\left[x\right]$ is the bin count of bin *x* in the zero-aligned histogram $H_{m}^{D}$ of the upper (M−m) bits of the magnitudes of residuals of the time series data D, and $\mathrm{supp}(H_{m}^{D})$, the support of $H_{m}^{D}$, is the set of bins in $H_{m}^{D}$ with nonzero bin counts.

Furthermore, let $\mathrm{supp}(H_{m}^{V})$ be partitioned into the following index sets

(8) $$A_{m,k}≜\mathrm{supp}\left(H_{m}^{V}\right)\cap \mathrm{dom}\left(T_{m,k}\right)$$

and

(9) $$B_{m,k}≜\mathrm{supp}\left(H_{m}^{V}\right)\backslash A_{m,k}$$

$A_{m,k}$ is the set of probability classes in the validation dataset with codes in the compression table. $T_{m,k}$ and bins in $H_{m}^{V}$ with nonzero bin counts are stored in hash tables, so $A_{m,k}$ is the set of keys that are common to both $T_{m,k}$ and $H_{m}^{V}$. The expression in Equation (7) can therefore be expanded as follows

(10) $$\sum_{x\in \mathrm{supp}(H_{m}^{V})}H_{m}^{V}\left[x\right]\phi \left(x;m,M,T_{m,k}\right)=\sum_{a\in A_{m,k}}H_{m}^{V}\left[a\right]\left(2+\mathrm{len}(T_{m,k}\left[a\right])+m\right)+\sum_{b\in B_{m,k}}H_{m}^{V}\left[b\right]\left(1+M\right)$$

However, the number of samples without codes in the compression table is simply the difference between the total number of samples and the samples with codes in the table, i.e.,

(11) $$\sum_{b\in B_{m,k}}H_{m}^{V}\left[b\right]=N-\sum_{a\in A_{m,k}}H_{m}^{V}\left[a\right]$$

Substituting the constraint in Equation (11) into Equation (10) and simplifying yields the following expression for efficiently evaluating Φ

(12) $$\Phi \left(V;m,M,T_{m,k}\right)=N\left(1+M\right)-\sum_{a\in A_{m,k}}H_{m}^{V}\left[a\right]\left(\lambda_{m}-\mathrm{len}\left(T_{m,k}\left[a\right]\right)\right)$$

where

(13) $$\lambda_{m}≜M-m-1$$

Equation (12) assumes that $H_{m}^{V}$ is given—for it to be significantly more efficient than Equation (2). $H_{m}^{V}$ may be computed naively by making an expensive O(N) scan through V, however, the method described below, which relies on the observation that $H_{m}^{V}$ is sparse, can be used to efficiently compute $H_{m}^{V}$ for m>0.

**Proposition** **1.** *The bin counts $H_{m+1}^{D}\left[x\right]$ of bin x in histogram $H_{m+1}^{D}$ can be computed from $H_{m}^{D}$ via the following recurrence relation, where the base case $H_{0}^{D}$ is constructed directly from the dataset D.*

(14) $$H_{m+1}^{D}\left[x\right]=\sum_{y∋x=\left\lfloor \frac{y}{2}\right\rfloor }H_{m}^{D}\left[y\right]\;0\leq m<M-1$$

**Proof.**  Bins *x* and *y* contain data that map to the same value with the transformations $\left\lfloor \left|\cdot \right|/2^{m+1}\right\rfloor$ and $\left\lfloor \left|\cdot \right|/2^{m}\right\rfloor$, respectively. Therefore, it is sufficient to show that, for any $z\in Z_{\geq 0}$,

(15) $$\left\lfloor \frac{z}{2^{m+1}}\right\rfloor =\left\lfloor \frac{\left\lfloor \frac{z}{2^{m}}\right\rfloor }{2}\right\rfloor$$

An intuitive but admittedly informal discussion for the more general constraint m≥0 is given here; however, a formal proof is provided in Appendix A.2. Any nonnegative integer *z* has a unique binary representation [31], in other words, for some M>0 and $b_{i}\in \left\{0,1\right\}$, $z=\sum_{i=0}^{M-1}b_{i}2^{i}$. Integer division of *z* by $2^{m+1}$ (the left-hand side (LHS) of Equation (15)) can be implemented with a right shift of the binary representation of *z* by m+1 bits [32]. However, a right shift by m+1 bits can be performed recursively, i.e., with a right shift by *m* bits followed by a right shift by one bit (the right hand side (RHS) of Equation (15)). Therefore, both sides of Equation (15) are equal. □

The following provides justification for the claim that Algorithm 2 may be used to compute compression tables from arbitrarily large streaming datasets:
**Algorithm 2** Fast hyperparameter optimization and Huffman table construction.**Input:**$S,M,k_{1},k_{2}$**Output:**$T_{m^{*},k^{*}}$1:{T,V}←partition(S)2:$H_{0}^{T}\leftarrow \mathrm{histogram}\left({\left|\Delta T\right|}_{n}\right)$3:$H_{0}^{V}\leftarrow \mathrm{histogram}\left({\left|\Delta V\right|}_{n}\right)$4:m←05:$N\leftarrow \left|V\right|$6:Φ←{}7:$\Phi_{\max}\leftarrow N\left(1+M\right)$8:**do**9:$\;\lambda_{m}\leftarrow M-m-1$10:  HmTs←sort(HmT,‘descending’)11:  **for** $k\leftarrow k_{1}$ **to** $k_{2}$ **do**12:    $\Phi \left[m,k\right]\leftarrow \Phi_{\max}$13:    Tm,k←huffman(HmTs[0:k))14:    **for** **all** $a\in \mathrm{dom}(T_{m,k})$ **do**
15:      **if** $a\in \mathrm{supp}(H_{m}^{V})$ **then**16:       $\Phi \left[m,k\right]\leftarrow \Phi \left[m,k\right]-H_{m}^{V}\left[a\right]\left(\lambda_{m}-\mathrm{len}\left(T_{m,k}\left[a\right]\right)\right)$17:      **end** **if**
18:    **end** **for**
19:  **end** **for**
20:$\;H_{m+1}^{T}\overset{\left(14\right)}{⟵}H_{m}^{T}$21:$\;H_{m+1}^{V}\overset{\left(14\right)}{⟵}H_{m}^{V}$22:  m←m+123:**while**m<M24:$m^{*},k^{*}\leftarrow {arg min}_{m,k}\Phi \left[m,k\right],\;0\leq m<M,\;k_{1}\leq k\leq k_{2}$25:Tm*,k*←huffman(Hm*Ts[0:k*))***Comments:** (1)*${\left|\Delta D\right|}_{n}$*is the sequence of magnitudes of the first backward difference of*D*; (2)*$g[n_{1}:n_{2})$*denotes the sequence*$g\left[n_{1}\right],g\left[n_{1}+1\right],\ldots ,g\left[n_{2}-1\right]$*; and (3)*$H_{m+1}^{D}$*is computed with the recurrence relation in Equation *(*14*)

1. Algorithm 2 does not invoke the encoder because only the knowledge of a sample’s probability class, but not its actual code, is required to compute its code length, which implies that number of primitive operations within each loop is reduced.
2. There is no need to explicitly compute the code lengths for samples whose probability classes are not in the compression table because they have already been implicitly accounted for by the first term in Equation (12).
3. In Algorithm 2, the innermost *for-all-loop* over the *k* entries in the compression table replaces the *for-loop* over the *N* samples in the validation dataset with the naive implementation. The table size is several orders of magnitude smaller than the size of the validation data. Moreover, the predicates in the innermost *for-all-loop* and its associated *if-statement* may be interchanged if $\left|\mathrm{supp}(H_{m}^{V})\right|<k$, i.e., the size of the histogram is smaller than the size of the table, so that the cost of evaluating Φ[m,k] is O(K), where $K≜\min(k,\left|\mathrm{supp}(H_{m}^{V})\right|)$ (in contrast to the O(N) cost with the naive method). For instance, the worst case cost of Φ given $T_{3,5}$ in the simulated example in Figure 3 is proportional to 5—whether the size of the validation dataset is $10^{3}$ or $10^{100}$ is irrelevant.
4. $\lambda_{m}-\mathrm{len}\left(T_{m,k}\left[a\right]\right)$ is the number of bits saved by compressing a sample within the probability class *a*. However, instead of computing those values one-at-a-time, the histogram is used to compute the total number of bits saved by all samples within the probability class *a*.
5. Huffman tables for sorted data can be computed in linear time, and the size of the table is typically very small, so the cost of computing $T_{m,k}$ is low.
6. $H_{m}^{D}$ is independent of *k*, so $H_{m^{\prime }}^{D}$ is only computed once to evaluate Φ with $\left\{m^{\prime },k_{1}\right\}$, $\left\{m^{\prime },k_{1}+1\right\},\ldots ,\left\{m^{\prime },k_{2}\right\}$.
7. $H_{m+1}^{D}$ is computed with Equation (14) in linear time by making a single pass through $H_{m}^{D}$. Furthermore, $H_{m}^{D}$ is sparse and $\left|\mathrm{supp}(H_{m}^{D})\right|$ typically decays exponentially in *m* by a factor of 2 (both can be observed for m∈{0,1} in Figure 2). Therefore, computing $H_{m}^{D}$ with Equation (14) becomes progressively cheaper with increasing *m*.
8. The cost of the *do-while-loop*, which executes the brute-force search, is decoupled from the size of the data (and becomes fixed) once the number of nonzero bins in $H_{0}^{T}$ and $H_{0}^{V}$ converge because the loop only depends on S through $H_{0}^{T}$ and $H_{0}^{V}$. One important consequence of this result (which was not exploited in this work) is that the algorithm can be made to be very memory efficient because there is no need to explicitly store the raw data. $H_{0}^{T}$ may be constructed by incrementally updating the histogram for the first *N* samples received from the embedded system, while $H_{0}^{V}$ may be similarly constructed from the latter *N* samples. Moreover, the cost of computing the histograms in this manner is negligible because it is spread out over time.

### 2.2. Hardware

**Figure 4:**
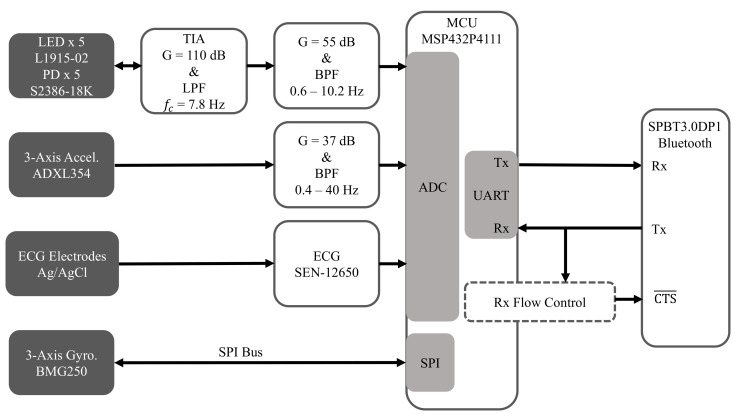
Hardware architecture for acquiring ECG, SCG, GCG, and rPPG.

Figure 4 is a high-level overview of the hardware architecture of the embedded system, which is comprised of an MCU that interfaces with custom and off-the-shelf analog front-ends for acquiring the multimodal physiological signals described below.

#### 2.2.1. Electrocardiogram (ECG)

The SEN-12650 ECG module (Sparkfun Electronics, Niwot, CO, USA) that is based on the AD8232 ECG front-end (Analog Devices Inc., Norwood, MA, USA) was used for recording ECG. Sparkfun has open-sourced the design of the SEN-12650, which we will fully incorporate into a more compact single-board implementation in the future.

#### 2.2.2. Seismocardiogram (SCG)

SCG is a measurement of chest-wall vibrations in response to cardiac contraction and the ejection of blood at the aortic valve [33,34], and it is typically used for proximal pulse detection. It has been shown that it can be measured with an accelerometer placed on the sternum [35,36]. In this work, the low-noise ADXL354 3-axis accelerometer (Analog Devices Inc., Norwood, MA, USA) was used to record SCG. The signal amplitudes are typically low, so a custom gain circuit is used to amplify the signal so that the ADC input range can be better utilized.

#### 2.2.3. Gyrocardiogram (GCG)

The ultra-low noise BMG250 (Robert Bosch GmbH, Gerlingen, Germany) 3-axis gyroscope attached to the sternum was used to record GCG data, which measures angular chest-wall vibrations in response to heartbeats [37]. It has been shown that cardiac timing estimation accuracy can be improved by using GCG, along with SCG, for proximal pulse detection [38,39].

#### 2.2.4. Reflectance Mode Photoplethysmogram (rPPG)

rPPG is used for distal pulse detection in the context of PTT estimation. In reflectance mode, the photodiode (PD) detects back-scattered or reflected light from tissue, bone or blood vessels, in contrast to transmissive mode PPG, where light is transmitted from a light emitting diode (LED) through the body to the PD located opposite the LED [26]. Two channels of rPPG were recorded at the sternum and one channel was recorded at the finger with a custom PPG circuit developed in our lab. For each chest PPG channel, two L1915-02 infrared LEDs (Hamamatsu Photonics K.K., Hamamatsu, Japan) were connected in series and two S2386-18K PDs (Hamamatsu Photonics K.K., Hamamatsu, Japan) were connected in parallel to boost the signal-to-noise ratio (SNR), which is typically low when rPPG signals are measured at poorly perfused anatomical regions of the body. In contrast, only a single LED-PD pair was used to record rPPG at the finger because rPPG waveforms recorded there have a much higher signal quality.

#### 2.2.5. Microcontroller

The MSP432P4111 MCU (Texas Instruments Inc., Dallas, TX, USA) with a single-core 48 MHz processor was used because of its ultra-low power consumption, as well as its relatively large 256 KB RAM, which provides sufficient room for implementing an ACK-based lossless communications protocol and for storing the compression tables. In addition, it is equipped with a 14-bit analog-to-digital converter (ADC), which was used to sample SCG, PPG, and ECG data, and an SPI module for interfacing with the BMG250 gyroscope. It also has a comparator module that was used for the UART flow control system described below.

#### 2.2.6. Bluetooth Module

The SPBT3.0DP1 (STMicroelectronics, Geneva, Switzerland) classic Bluetooth module with enhanced data rate was used because of its relatively high sustained data rate of 450,000 bit/s and its easy-to-use serial pass-through capabilities, which greatly simplified prototyping. However, future designs will utilize a more modern Bluetooth transceiver, such as the CC2564 dual-mode Bluetooth transceiver (Texas Instruments Inc., Dallas, TX, USA) to enhance design flexibility.

#### 2.2.7. UART Flow Control

One major issue that had to be addressed during prototyping was the severe data loss that occurred whenever multi-byte packets (required for messages, data acknowledgments, and compression tables) were transmitted from the phone to the embedded system through the Bluetooth module over UART at baud rates exceeding 115,200 bit/s. The standard UART flow control mechanism, which involves raising the $\bar{\mathrm{CTS}}$ flow control line in an interrupt service routine (ISR), is inadequate because the underlying problem is related to the MCU’s relatively high interrupt latency (meaning that the CPU cannot react quickly to incoming data) when the data rate is high, coupled with the asynchronous nature of UART communications, but not due to insufficient bandwidth to satisfy the very modest application data rate of less than 2.5 kB/s. As a concrete example, even if the required bandwidth is just 2 B/s, but both bytes are sent in rapid succession, then the second byte overwrites the first before it is copied from the receive buffer or before the Bluetooth module responds to the $\bar{\mathrm{CTS}}$ line that is raised in the ISR.

**Figure 5:**
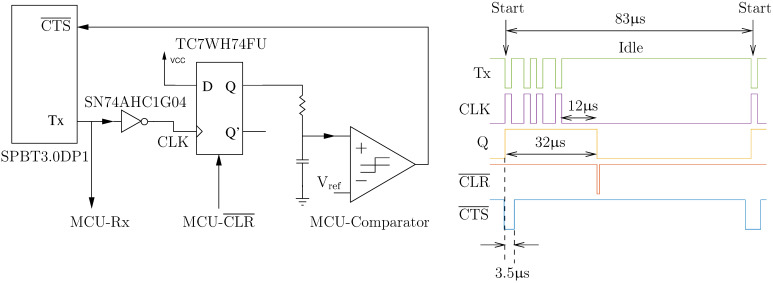
UART flow control circuit with a timing diagram (recorded with a logic analyzer) of a single-byte frame of a seven-byte acknowledgment packet.

The hybrid hardware–software flow control solution that was developed in this work is illustrated in Figure 5, and it makes use of the knowledge that UART data lines idle high and that a high-to-low transition (known as a start bit) indicates the beginning of a new byte. The start bit is detected by the D-type flip-flop (after inversion) and the circuit autonomously asserts the $\bar{\mathrm{CTS}}$ line, without any software intervention, by propagating the high latched value to the $\bar{\mathrm{CTS}}$ line after a Bluetooth-device-specific delay implemented with a combination of a first-order RC circuit and a comparator. Data flow from the Bluetooth module can then subsequently be re-enabled at a leisurely pace when the MCU is ready to receive more data by applying a reset pulse ($\bar{\mathrm{CLR}}$) to the flip-flop. It should be noted that the time between the start bit and when $\bar{\mathrm{CTS}}$ is asserted was empirically determined to be 3.5 μs (with baud rate = 460,800 bit/s) by trial-and-error because it was not specified in the datasheet. In addition, a hysteresis effect on the comparator was used to add a 50 μs delay between the reset pulse and when $\bar{\mathrm{CTS}}$ is deasserted so that the MCU can service other time-critical code.

### 2.3. Software

**Figure 6:**
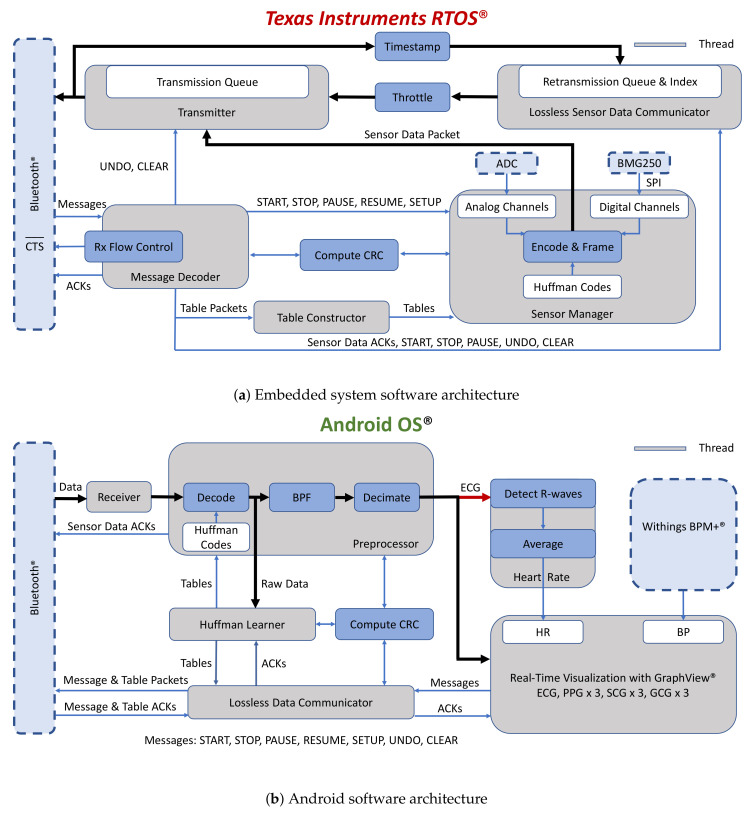
Software architecture for real-time data acquisition of physiological data and lossless data communication over Bluetooth.

Figure 6a provides a high-level illustration of the software architecture for the MCU. The firmware was written in C++ with Texas Instruments’ real-time operating system (RTOS), as opposed to a bare-metal approach, to reduce implementation complexity. An ACK-based lossless data communications method was implemented, where transmitted sensor data are buffered and retransmitted if an acknowledgment for the sensor data is not received before a one-second timeout. The Index provides fast and efficient O(1) access to the packets in the retransmission queue, so that they can be efficiently removed when ACKs are received. The system also acknowledges messages (e.g., START and STOP) and compression tables. While 32-bit cyclic redundancy check (CRC) codes were used for error detection for sensor data and compression tables, 16-bit CRC codes were used to validate messages and requests from the smartphone and responses to those requests. Retransmissions of lost packets are limited when the embedded system is actively sampling data, thereby implicitly placing more importance on new data.

Figure 6b is a simplified block diagram of the Android application, running on a low-cost Pixel 3a XL smartphone (Google Inc., Mountain View, CA, USA), which was written in Java and Kotlin. Raw data are shared with the thread responsible for computing the compression tables before they are downsampled for visualization with the GraphView library. The application also interfaces with the BPM+ Android application (Withings, Issy-les-Moulineaux, France) so that the BP measurements made with the BPM+ device can be conveniently shared with our application. These measurements will be used as the gold standard for experiments that will be made with our system. The application saves sensor data, compression tables, and metadata to Google Drive for post-processing.

The desktop application was also written in Java and Kotlin but with the JavaFX graphical user interface development framework. The application is used for offline decompression of data, initial post-processing, and for exporting compression codes for initializing the compression tables on the smartphone and the embedded system.

The back-end server was implemented in Python with Google App Engine and Google Datastore. The server’s functionality is currently limited to generating globally unique subject IDs and for storing de-identified subject metadata (e.g., age and weight), but may be used to compute PTT and BP in future.

### 2.4. Subject Demographics

Six healthy subjects (gender: four males and two females with mean ± std: age, 27.5±5.8 years; height, 171.8±12.2 cm; and weight, 72.4±15.8 kg) were properly consented before participating in the protocol approved by Georgia Institute of Technology’s Institutional Review Board. Data from the first subject were only used to initialize the compression tables for one of the experiments in the main experimental protocol that involved the other five subjects.

### 2.5. Measurements

There were 50 samples of data for each channel in every packet. For uncompressed data, the number of bits per sample was 15 bits for the seven channels of SCG, ECG, and rPPG and 17 bits for each of the three GCG channels (with the raw data indicator included in the calculation). Furthermore, the size of the header and trailer was 19 B for each packet. Therefore, the size of each uncompressed packet was 50×(15×7+17×3)/8+19= 994 B.

#### 2.5.1. Data Acquisition at below the Theoretical Channel Capacity

Data recorded at a 1600 Hz sampling rate were used to demonstrate the plug-and-play capability of the proposed compression method, which enables sensors that have not previously been characterized to be interfaced to the embedded system. The initially empty compression tables are then updated after the compression tables have been computed by the smartphone from data recorded in real time. Subsequent samples are encoded using the compression tables, which reduces the bandwidth and power consumption of the Bluetooth radios. The maximum required bandwidth in this mode is 994×1600/50≈ 32 kB/s, which is less than the maximum channel capacity of 45 kB/s.

#### 2.5.2. Data Acquisition at above the Theoretical Channel Capacity

The maximum required bandwidth for data sampled at 3200 Hz is 994×3200/50≈ 64 kB/s, which is significantly higher than the channel capacity. This implies that data sampled at this rate cannot be transmitted in its raw form. Therefore, the compression tables were initialized with Huffman codes computed from data sampled at 1600 Hz from a subject who did not participate in the experimental protocol described below, which reduced the bandwidth to less than the theoretical channel capacity. The compression tables were subsequently updated once the smartphone was done computing tables from newly recorded data.

#### 2.5.3. Experimental Protocol

**Figure 7:**
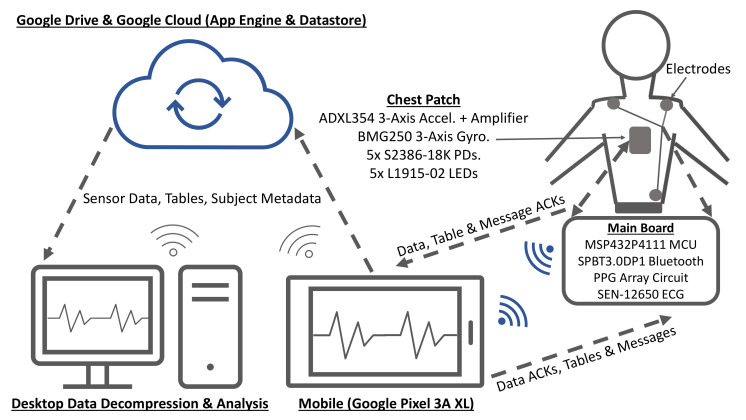
Block diagram for the experimental setup.

The experimental setup is shown in Figure 7 with subjects asked to place and hold the chest patch on their sternum for the entire duration of each experiment. The ECG electrodes were placed on the torso in a Lead I configuration. The search parameters $k_{1}$ and $k_{2}$ for the compression table size were chosen to be 10 and 30, respectively. The compression tables were computed on the smartphone with Algorithm 2 from the first two minutes of data at each of the sampling rates and then the tables were transmitted to the embedded system and subsequently used to compress data recorded for another three minutes. Thus, the total duration for each experiment was approximately five minutes. It should be noted that compression tables were simultaneously computed from all of the residuals in the training data and saved for use as the gold standard. The sensor data packets are explicitly tagged with a compression table ID, which greatly reduces the cost of synchronization of the tables on the smartphone with the MCU (the ID of tables computed online is 1). The seemingly arbitrary sampling rates of 1600 and 3200 Hz were selected because they are the two highest native sampling rates supported by the BMG250 gyroscope. In addition, although the 3200 Hz sampling rate is higher than typical sampling rates for PTT applications, the performance of the proposed algorithm was evaluated at this frequency for the following reason. The design of the wearable is likely to evolve towards the use of off-the-shelf analog front-ends, such as the AFE4900 (Texas Instruments Inc., Dallas, TX, USA) and the ADXL355 (Analog Devices Inc., Norwood, MA, USA), which have ADC resolutions of 24- and 20-bits, respectively (higher than the 14- and 16-bit resolutions of waveforms recorded in this work). Therefore, the higher 3200 Hz sampling rate was used to simulate the higher average data rate that would be needed for future implementations.

### 2.6. Post-Processing

First, the received packets were rearranged because they were not necessarily received in the correct order. Second, the packets with compression table ID 1 were unrolled with the sensor data grouped according to their respective channels. The compression ratios were computed on data with ID 1 with the compression tables for the proposed and standard methods.

Duplicate sensor data packets on the receiver may be due to: (1) an insufficiently large packet acknowledgment timeout parameter after which a packet is retransmitted; and (2) a corrupted data acknowledgment packet, which also results in a retransmission. A generous timeout value of one second was specified for the application—although acknowledgments are typically received in less than 100 ms. Therefore, a duplicate sensor data packet indicates that the acknowledgment packet was dropped due to packet corruption, and this property was used to evaluate the efficacy of the proposed flow control method.

## 3. Results and Discussion

### 3.1. Compression

**Figure 8:**
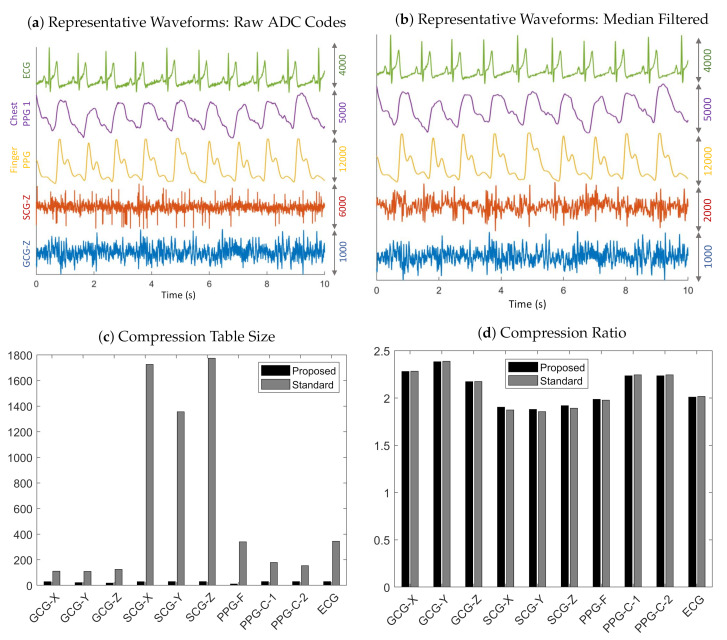
Representative results of data recorded from Subject 4 at 3200 Hz. (**a**) Ten-second trace of raw ADC codes for ECG, chest PPG, finger PPG, SCG-Z, and GCG-Z data. (**b**) Filtering with a seventh-order median filter reveals the underlying signal in the electronic noise corrupted raw SCG-Z signal. (**c**) Compression table sizes for the standard and proposed methods. (**d**) Compression ratios for the standard and the proposed methods.

The plots in Figure 8a are representative waveforms of the raw ADC codes for GCG-Z, SCG-Z, finger PPG, chest PPG 1, and ECG recorded from Subject 4 at 3200 Hz. Some interference was present in the SCG-Z signal (in the form of impulsive noise), which was likely due to the unshielded cables used in the experiments. The amplitudes and signal quality of the GCG-Z and SCG-Z are rather low, but the ECG, chest PPG, and finger PPG waveforms all exhibit markedly higher signal quality. The finger PPG signal is very clean and has such a high dynamic range that the waveform is close to saturating beat-to-beat. The amplitudes of the chest PPG and ECG are also quite strong, with clean signal features visible.

Table 1 reveals that our proposed method enabled a table size decrease of 67–99% across all sensing modalities and sampling rates, thereby optimizing the MCU memory consumption. In particular, the average table size for the standard method is 1728 for SCG-Z data recorded at 3200 Hz, which demonstrates why it is impractical for memory-constrained embedded systems because it would require, at a bare minimum, 19 kB of memory for the SCG-Z channel alone (11 B for each table entry: 2 B for the key, 4 B for the code, 1 B for the code length, and 4 B for the pointer to the next entry in the hash table bucket). In contrast, the worst case memory usage, with the 11 B memory allocation per table entry analysis above, is only 330 B for our proposed method. The reason that the tables sizes are much larger for SCG data in comparison to the other sensing modalities is likely due to the impulsive noise, which violates the underlying assumption of differential pulse code modulation that adjacent samples are highly correlated in naturally occurring signals. The impulse-like QRS complex in the ECG similarly causes its table sizes (with the standard method) to be significantly larger than those of other sensing modalities.

Another interesting observation that can be made from the results in Table 1 is that the hyperparameter search algorithm does not greedily always select a table of size $k_{2}$ (the specified maximum table size of 30), which may be due to either of the following reasons: (1) the trivial case when the requested table size is larger than possible, i.e., $k_{2}>\left|\mathrm{supp}(H_{m^{*}}^{T})\right|$; and (2) $\Phi \left[m^{*},k_{2}\right]\geq \Phi \left[m^{*},k^{*}\right]$. During post-processing, we only had access to $\mathrm{supp}(H_{m}^{T})$ for m=0 (in the form of the keys of the table for the standard method). However, for $m^{*}\geq 0$, Proposition A1 was used to check if the trivial case could definitely be ruled out, which was indeed the case for 64 out of 65 times when the selected table size was less than $k_{2}$. $\Phi \left[m^{*},k_{2}\right]\geq \Phi \left[m^{*},k^{*}\right]$ arises when increasing the table size results in either a tie or an increase in the total number of bits. The latter may occur as a consequence of the prefix-free property of Huffman codes, which states that no valid code is a preceding subsequence of another. To maintain this invariant, the code lengths of an initial set of probability classes may increase as the table size is increased. This may be seen in the simulated example in Figure 3 where the codes for the probability Classes 1 and 2 would have been 0 and 1 (or possibly flipped), regardless of their relative frequencies, if they had been the only entries in the table. However, the code length for Class 2 is two because of the other probability classes added to the table. If the relative frequencies of the additional classes in the validation data are not consistent with the training data, for example if none of those additional table entries is present in the validation data, then the total number of bits increases due to the larger code length of samples in Class 2 that are not compensated for by gains from the other table entries. It should also be noted that code lengths of classes present in the validation data but not in the training data are of fixed length (1+M). Both observations demonstrate how, by splitting the data into training and validation datasets, the hyperparameter search algorithm selects codes that are more robust to noise than would be the case if the codes were generated without the validation data.

The main observation that can be drawn from the results in Table 2 is that the compression ratios obtained with the proposed method matches those of the standard approach—even with much smaller tables. In addition, the compression ratios for the GCG and chest PPG are, in general, larger than those of the finger PPG, ECG, and SCG, which suggests that impulsive signals and those with high dynamic range have lower compression ratios than signals with low amplitudes or slowly varying waveforms. It should be noted that the reported compression ratios for SCG, ECG, and PPG data are relative to the ADC’s 14-bit resolution, so the actual compression ratios are higher if one considers the two unused bits in the 16-bit representation of the raw ADC codes.

There was an across-the-board increase in compression ratios for the SCG, PPG, and ECG data when the sampling rate was increased from 1600 to 3200 Hz, which may be due to a decrease in the magnitude of residuals when the sampling rate is increased. However, this relationship is reversed for the GCG data. It is not immediately clear why, but one reasonable explanation is that the low signal levels of the GCG makes the residuals more susceptible to being dominated by random noise. Nevertheless, the changes in compression ratios in both directions demonstrates the dependence of the performance of the compression tables on the sampling rate amongst other factors, which illustrates why learning the compression tables in real-time is superior to computing them offline.

**Figure 9:**
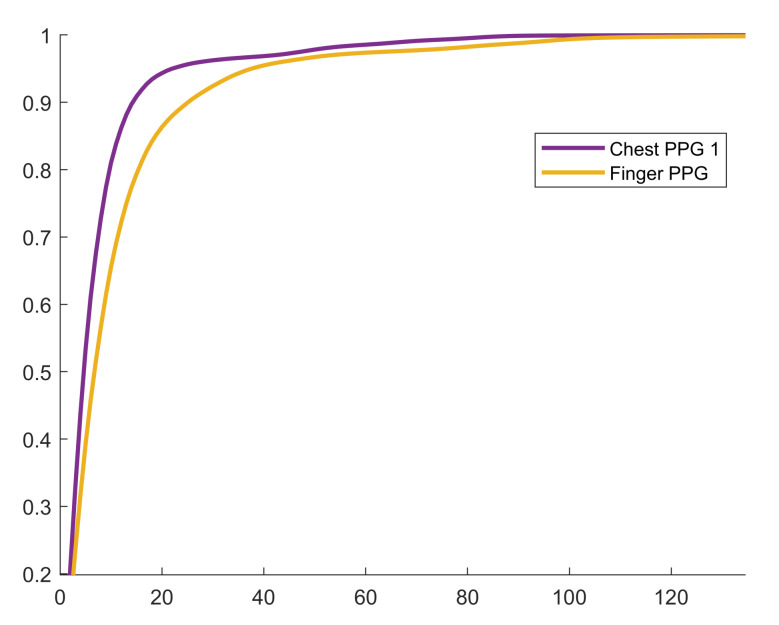
Empirical cumulative distribution function for Subject 4 at 3200 Hz.

We present all the chosen values for $m^{*}$, the selected bin width parameter, as opposed to the mean ± std statistics because outliers hide important trends in the result. Specifically, the high degree of intra-channel consistency and inter-channel variance demonstrate the hyperparameter search algorithm’s ability to adapt to the underlying signal. For example, for the GCG-X channel, $m^{*}=1$ for all subjects. Furthermore, the mode was selected at least 50% of the time for nine out of ten channels, and at least 70% of the time for six out of ten channels. Another key observation is that $m^{*}$ is typically larger for impulsive signals or those with a high dynamic range, which explains the outlier in Table 3 where $m^{*}=3$ for PPG-C-1 recorded at 1600 Hz from Subject 1 that was saturating beat-to-beat. This behavior was not observed for other measurements, so it was likely as a consequence of the contact pressure applied by the subject during that experiment. In Figure 9, the cumulative distribution function (CDF) of the finger PPG lies below the chest PPG’s, which shows that the distribution of residuals for the finger PPG is more spread out, and that causes the algorithm to select a larger bin width to compensate for the spread. The compression table of the finger PPG recorded from Subject 4 has 11 and 340 entries for the proposed and standard methods, respectively, although the compression ratios obtained with both methods are practically identical. The reason is that the table is able to encode 88 unique residual magnitudes given the bin width of eight and the table size of 11. The CDF at 88>0.98, which demonstrates how a very compact compression table may be used to achieve a very high code density.

The combined duration for executing the hyperparameter search algorithm for all ten channels was 1050±158 ms and 2012±151 ms for the 1600 Hz and 3200 Hz sampling rates, respectively. Although not explicitly shown in Algorithm 2, the hyperparameter search algorithm terminates early once $k_{1}>\left|\mathrm{supp}(H_{m}^{T})\right|$, i.e., the minimum acceptable table size is greater than the number of histogram bins, so we also recorded the combined number of candidate {m,k} pairs that Φ was evaluated with across all channels, which was 1254±103 and 1305±47, for the 1600 and 3200 Hz sampling rates, respectively. The short duration required to compute the tables and the large number of searches, which indicates that the algorithm did not terminate only after a few iterations, demonstrates the performance of the proposed hyperparameter search method even with large datasets of 192,000 and 384,000 samples for the 1600 and 3200 Hz sampling rates, respectively. Moreover, the cost of constructing $H_{0}^{T}$ and $H_{0}^{V}$ likely dominates the cost of the actual search; therefore, the algorithm can be made to run more quickly by constructing them with the incremental approach described in Section 2.1.2 for larger datasets.

### 3.2. UART Flow Control

There were no duplicate packets among the 160,550 correctly received sensor data packets across all subjects and experiments. Therefore, every seven-byte acknowledgment packet that was sent by the smartphone for every correctly received data packet was correctly received by the embedded system. The computed empirical error rate for data sent in the path from the smartphone to the embedded system was identically 0%, which demonstrates the effectiveness of the proposed flow control method. It should be noted that the system has been successfully tested at an even higher baud rate of 921,600 bit/s. However, because the maximum sustained data rate of the SPBT3.0DP1 Bluetooth module is 450,000 bit/s, the closest standard baud rate of 460,800 bit/s was used.

### 3.3. Limitations

In this work, the compression tables on the MCU were unconditionally updated after learning them on the smartphone. However, there is no guarantee that the new table will, at the very least, perform as well as the previous table (unless in the trivial case when the previous table is empty). One way to guard against updating a compression table with an inferior one is by evaluating both tables with data recorded after the one used to compute the table (this may also be done by partitioning the data into training, validation, and test datasets, with the test data used for the assessment). A decision can then be made as to whether the compression tables should be updated based on the outcome of the evaluation.

The lossless communications method developed in this work is limited to correcting random packet loss, and is not well-suited to more significant communication failures like long-term Bluetooth connection loss because the MCU’s 256 KB RAM only provides a few seconds of buffering capacity. In practice, however, this constraint has not been an issue and we have successfully tested the prototype by continuously streaming data for two hours.

The choice of a D-type flip-flop, as opposed to a more appropriate D-type latch, was dictated by availability (at the time of prototyping) in limited quantities required for device development. Nevertheless, for this application, a D-type flip-flop can emulate a D-type latch because the only value that needs to be latched onto is a high, and this can be done by permanently connecting the D input to high. It should also be noted that only the following discrete components are actually required: a D-type latch, a resistor, and a capacitor. That is because most MCUs, including the MSP432P4111, have on-board comparators and there are D-type latches with inverting clock inputs, which eliminates the need for a discrete comparator and an inverter.

Finally, the performance of the approach in persons with cardiovascular diseases and arrhythmias must be characterized to understand generalizability of the methods. Specifically, the impact of arrhythmias on the compression algorithm will need to be studied in future work. Broadly speaking, the signals measured in this work have been demonstrated to be of high quality even in patients with severe left ventricular dysfunction, i.e., patients with advanced heart failure [40,41], and thus the potential for this work to be applicable to such populations holds merit.

## 4. Conclusions

We developed a prototype of an end-to-end system for conveniently recording high throughput physiological data that will be used for monitoring BP in a mobile setting. The system is comprised of custom and off-the-shelf analog front-ends for recording ECG, rPPG, SCG, and GCG; a Bluetooth module for transmitting acquired data to a Bluetooth-enabled Android smartphone; and a software suite with MCU firmware, an Android application, a back-end web server, and desktop software. We also developed a computationally inexpensive source coding method that is based on Huffman coding and we showed that the proposed method can match the compression ratios obtained with the standard algorithm while requiring significantly less memory for storing compression tables. In addition, we implemented a UART flow control method that minimizes data loss due to the interrupt-latency of low-power MCUs. Future work will include developing a PCB with the components of the current breadboard prototype and performing field studies with the device for continuous ambulatory BP monitoring. We will also investigate other methods for increasing data acquisition efficiency such as only intermittently recording data at a high sampling rate for BP estimation and recording data at a reduced sampling rate for the rest of the time. The data recorded at the lower sampling rate may then be used to estimate the subject’s physiological state (e.g., heart rate), and a significant state change may serve as an additional trigger for recording high throughput data for BP estimation. The software and hardware designs developed in this work will be open-sourced to further promote PTT research.

## Figures and Tables

**Table 1:** Compression table size.

	1600 Hz		3200 Hz	
**Sensor**	**Proposed**	**Standard**	**Proposed**	**Standard**
GCG-X	23±1	104±35	28±1	113±9
GCG-Y	21±6	113±46	20±5	120±26
GCG-Z	22±1	127±45	28±5	121±10
SCG-X	27±4	868±121	26±5	1630±75
SCG-Y	21±2	739±99	29±1	1566±300
SCG-Z	26±3	832±133	24±4	1728±72
PPG-F	23±6	195±37	18±9	358±121
PPG-C-1	26±8	228±118	30±0	181±39
PPG-C-2	25±6	241±153	27±3	175±36
ECG	28±1	398±218	29±1	420±68

**Table 2:** Compression ratio.

	1600 Hz		3200 Hz	
**Sensor**	**Proposed**	**Standard**	**Proposed**	**Standard**
GCG-X	2.37±0.04	2.37±0.04	2.28±0.01	2.28±0.01
GCG-Y	2.43±0.07	2.43±0.07	2.36±0.03	2.37±0.03
GCG-Z	2.36±0.03	2.36±0.02	2.18±0.01	2.18±0.01
SCG-X	1.85±0.27	1.84±0.27	1.95±0.19	1.91±0.18
SCG-Y	1.87±0.22	1.86±0.22	1.96±0.13	1.93±0.13
SCG-Z	1.89±0.22	1.87±0.22	1.98±0.16	1.95±0.16
PPG-F	1.81±0.04	1.80±0.06	2.02±0.05	2.01±0.06
PPG-C-1	2.18±0.18	2.18±0.17	2.28±0.03	2.28±0.03
PPG-C-2	2.13±0.17	2.13±0.17	2.25±0.02	2.25±0.02
ECG	1.80±0.09	1.79±0.10	1.93±0.08	1.93±0.08

**Table 3:** Bin width parameter $m^{*}$ for each of the five subjects.

	1600 Hz					3200 Hz				
**Sensor**	**1**	**2**	**3**	**4**	**5**	**1**	**2**	**3**	**4**	**5**
GCG-X	1	1	1	1	1	1	1	1	1	1
GCG-Y	2	1	1	1	1	2	1	1	1	2
GCG-Z	1	1	1	1	1	1	1	1	2	1
SCG-X	2	2	0	2	1	2	2	0	1	0
SCG-Y	2	2	1	2	1	1	1	0	1	0
SCG-Z	2	2	1	2	1	2	2	1	1	1
PPG-F	2	2	3	2	2	1	1	3	3	3
PPG-C-1	3	0	0	0	0	0	0	0	1	0
PPG-C-2	2	0	1	1	1	1	1	1	1	0
ECG	2	2	2	2	2	1	2	2	1	2

## Appendix A

### Appendix A.1. Table Size Check

**Proposition** **A1.**
*If $k_{2}$ (i.e., the upper limit of the table size search range) is less than or equal to the number of bins with nonzero bin counts in the histogram of bin width = 1, divided by the selected bin width, then $k_{2}$ is less than or equal to the number of bins with nonzero bin counts in the histogram of the selected bin width, i.e.,*

(A1) $$k_{2}\leq \frac{\left|\mathrm{supp}(H_{0}^{T})\right|}{2^{m^{*}}}\Rightarrow k_{2}\leq \left|\mathrm{supp}(H_{m^{*}}^{T})\right|\;m^{*}\in Z_{\geq 0}$$

**Proof.** Proposition A1 is vacuously true for $m^{*}=0$, so we focus on the proof for $m^{*}>0$. The following recurrence relation can be inferred from the recurrence relation in Proposition 1

(A2) $$\left|\mathrm{supp}(H_{m}^{D})\right|\geq \frac{\left|\mathrm{supp}(H_{m-1}^{D})\right|}{2}\;\forall m>0$$

from which it can be shown by induction that

(A3) $$\left|\mathrm{supp}(H_{m}^{D})\right|\geq \frac{\left|\mathrm{supp}(H_{0}^{D})\right|}{2^{m}}$$

therefore,

(A4) $$k>\left|\mathrm{supp}(H_{m}^{D})\right|\Rightarrow k>\frac{\left|\mathrm{supp}(H_{0}^{D})\right|}{2^{m}}$$

and by contraposition (a⇒b⇔¬b⇒¬a),

(A5) $$k\leq \frac{\left|\mathrm{supp}(H_{0}^{D})\right|}{2^{m}}\Rightarrow k\leq \left|\mathrm{supp}(H_{m}^{D})\right|$$

thus,

(A6) $$k_{2}\leq \frac{\left|\mathrm{supp}(H_{0}^{T})\right|}{2^{m^{*}}}\Rightarrow k_{2}\leq \left|\mathrm{supp}(H_{m^{*}}^{T})\right|$$

□

### Appendix A.2. Alternative Proof for Proposition 1

**Proof.** Bins *x* and *y* contain data that map to the same value with the transformations $\left\lfloor \left|\cdot \right|/2^{m+1}\right\rfloor$ and $\left\lfloor \left|\cdot \right|/2^{m}\right\rfloor$, respectively. Therefore, it is sufficient to show that for any $z\in Z_{\geq 0}$

(A7) $$\left\lfloor \frac{z}{2^{m+1}}\right\rfloor =\left\lfloor \frac{\left\lfloor \frac{z}{2^{m}}\right\rfloor }{2}\right\rfloor$$

Any nonnegative integer *z* has a unique binary representation [31], i.e., for some M>0 and $b_{i}\in \left\{0,1\right\}$

(A8) $$z=\sum_{i=0}^{M-1}b_{i}2^{i}$$

Therefore,

(A9) $$\begin{array}{cc} \frac{z}{2^{m}} & =\sum_{i=0}^{M-1}b_{i}2^{i-m} \end{array}$$

(A10) $$\begin{array}{cc} & =\sum_{i=0}^{m-1}b_{i}2^{i-m}+\sum_{j=m}^{M-1}bj2^{j-m} \end{array}$$

but

(A11) $$\begin{array}{ccc} 0 & \leq & \sum_{i=0}^{m-1}b_{i}2^{i-m} \end{array}$$

(A12) $$\begin{array}{ccc} & \leq & \sum_{i=0}^{m-1}2^{i-m} \end{array}$$

(A13) $$\begin{array}{ccc} & = & \sum_{k=1}^{m}2^{-k} \end{array}$$

(A14) $$\begin{array}{ll} & =1-{\left(\frac{1}{2}\right)}^{m}\;(\mathrm{via}\;(\mathrm{Geometric}\;\mathrm{series}\;\mathrm{sum})) \end{array}$$

(A15) $$\begin{array}{ll} & <1\;\mathrm{for}\;0\leq m<\infty \end{array}$$

It should be noted that k≜−(i−m) in Equation (A13). Furthermore, $\left(\sum_{j=m}^{M-1}b_{j}2^{j-m}\right)\in Z_{\geq 0}$ for 0≤m<M, which implies that the fractional component of Equation (A10) is $\sum_{i=0}^{m-1}b_{i}2^{i-m}$, i.e.,

(A16) $$\left\lfloor \frac{z}{2^{m}}\right\rfloor =\sum_{j=m}^{M-1}b_{j}2^{j-m}$$

substituting m+1 for *m* in Equation (A16) implies that the LHS of Equation (A7) is given by

(A17) $$\left\lfloor \frac{z}{2^{m+1}}\right\rfloor =\sum_{j=m+1}^{M-1}b_{j}2^{j-(m+1)}$$

for the RHS of Equation (A7),

(A18) $$\frac{\left\lfloor \frac{z}{2^{m}}\right\rfloor }{2}=\frac{b_{m}}{2}+\sum_{j=m+1}^{M-1}b_{j}2^{j-(m+1)}$$

$\left(\sum_{j=m+1}^{M-1}b_{j}2^{j-(m+1)}\right)\in Z_{\geq 0}$ for 0≤m<M−1, which implies that

(A19) $$\left\lfloor \frac{\left\lfloor \frac{z}{2^{m}}\right\rfloor }{2}\right\rfloor =\sum_{j=m+1}^{M-1}b_{j}2^{j-(m+1)}$$

thus, both Equations (A17) and (A19) are equal, which proves the equivalence in Equation (A7) □

### Appendix A.3. Huffman Codes for ECG (Bin → Code (Counts))

$0\to 1111_{b}$ (5917), $1\to 0001_{b}$ (12078), $2\to 0000_{b}$ (12252), $3\to 0010_{b}$ (11607), 4→0011 (11272), $5\to 0101_{b}$ (10868), $6\to 0100_{b}$ (10947), $7\to 0110_{b}$ (10278), $8\to 0111_{b}$ (9537), $9\to 1001_{b}$ (9288), $10\to 1000_{b}$ (9291), $11\to 1010_{b}$ (8627), $12\to 1011_{b}$ (8235), $13\to 1101_{b}$ (7894), $14\to 1100_{b}$ (7965), $15\to 1110_{b}$ (7278).
